# Tuberculous bowel obstruction at a university teaching hospital in Northwestern Tanzania: a surgical experience with 118 cases

**DOI:** 10.1186/1749-7922-8-12

**Published:** 2013-03-16

**Authors:** Phillipo L Chalya, Mabula D Mchembe, Stephen E Mshana, Peter Rambau, Hyasinta Jaka, Joseph B Mabula

**Affiliations:** 1 Department of Surgery, Catholic University of Health and Allied Sciences-Bugando, Mwanza, Tanzania; 2 Department of Surgery, Muhimbili University of Health and Allied Sciences, Dar Es Salaam, Tanzania; 3 Department of Microbiology & Immunology, Catholic University of Health and Allied Sciences-Bugando, Mwanza, Tanzania; 4 Department of Pathology, Catholic University of Health and Allied Sciences-Bugando, Mwanza, Tanzania; 5 Department of Internal Medicine, Catholic University of Health and Allied Sciences-Bugando, Mwanza, Tanzania

**Keywords:** Bowel obstruction, Intestinal tuberculosis, Clinicopathological profile, Surgical management, Outcome, Tanzania

## Abstract

**Background:**

Bowel obstruction resulting from intestinal tuberculosis has been reported to be more prevalent in developing countries including Tanzania. This study was undertaken to describe the clinicopathological profile, surgical management and outcome of tuberculous intestinal obstruction in our local setting and to identify factors responsible for poor outcome among these patients.

**Methods:**

This was a prospective descriptive study of patients operated for tuberculous intestinal obstruction at Bugando Medical Centre (BMC) in northwestern Tanzania from April 2008 to March 2012. Ethical approval to conduct the study was obtained from relevant authorities. Statistical data analysis was performed using SPSS version 17.0.

**Results:**

A total of 118 patients with tuberculous intestinal obstruction were studied. The male to female ratio was 1.8: 1. The median age was 26 years (range 11-67 years). The modal age group was 21-30 years. Thirty-one (26.3%) patients had associated pulmonary tuberculosis and 25 (21.2%) patients were HIV positive with a median CD4+ count of 225 cells /μl. Small bowel strictures were the most common operative findings accounting for 72.9% of cases. The ileo-caecal region was the commonest area of involvement in 68 (57.6%) patients. The right hemicolectomy with ileo-transverse anastomosis was the most frequent surgical procedure performed in 66 (55.9%) patients. Postoperatively all the patients received antituberculous drugs for a period of one year. Postoperative complication rate was 37.3% and surgical site infection (SSI) was the most frequent complication in 42.8% of cases. HIV positivity and low CD4+ count were the main predictors of SSI (*p* < 0.001). The overall median length of hospital stay was 24 days. Patients who had postoperative complications stayed longer in the hospital and this was statistically significant (*p* = 0.011). Mortality rate was 28.8% and it was significantly associated with co-existing medical illness, delayed presentation, HIV positivity, low CD 4 count (<200 cells/μl), ASA class and presence of complications (*p* < 0.001). The follow up of patients was generally poor as more than fifty percent of patients were lost to follow up.

**Conclusion:**

Tuberculous bowel obstruction remains rampant in our environment and contributes significantly to high morbidity and mortality. The majority of patients present late when the disease becomes complicated. A high index of suspicion, proper evaluation and therapeutic trial in suspected patients is essential for an early diagnosis and timely definitive treatment, in order to decrease the morbidity and mortality associated with this disease.

## Background

Tuberculosis (TB), a communicable disease caused by *Mycobacterium tuberculosis*, is a common and major health problem worldwide [1]. Approximately one third of the world population is infected and about three millions die each year from this disease [1,2]. In developed countries the incidence of TB has become rare due to increased standards of living [3]. However, due to the influx of immigrants from third world countries, HIV infection and increasing use of Immunosuppressive therapy, the incidence of tuberculosis in developed countries is again on the rise [4]. In developing countries, tuberculosis remains the principal cause of death, probably due to ignorance, poverty, overcrowding, poor sanitation, malnutrition and coexistence with emergent diseases like AIDS [5]. Approximately 95% of new cases and 98% of deaths occur in developing countries [6,7].

Tuberculosis may involve any part of the body but abdomen is one of the commonest site of involvement after lungs [8]. In the abdomen, tuberculosis may affect the gastro-intestinal tract, peritoneum, lymph nodes and solid viscera.

The modes of infection of the intestinal tuberculosis include hematogenous spread from a primary lung focus that reactivates later or miliary tuberculosis, spread via lymphatics from infected nodes, ingestion of bacilli either from the sputum or from infected sources such as milk products, or by direct spread from adjacent organs [9].

Intestinal tuberculosis has usually one of the three main forms i.e. ulcerative, hypertrophic or ulcerohypertrophic, and fibrous stricturing form [10,11]. The disease can mimic various gastrointestinal disorders, particularly the inflammatory bowel disease, colonic malignancy, or other gastrointestinal infections [12].

It usually runs an indolent course and presents late with complications especially acute or sub-acute intestinal obstruction due to mass (tuberculoma) or stricture formation in small gut and ileocaecal region or gut perforation leading to peritonitis [13,14]. In spite of advances in medical imaging, the early diagnosis of abdominal tuberculosis is still a problem due to vague and non-specific symptoms and patients usually present when complications such as bowel obstruction or perforation had occurred [15]. The most common complication of abdominal tuberculosis is obstruction due to narrowing of the lumen by hyperplastic caecal tuberculosis, by strictures of the small intestine, which are commonly multiple, or by adhesions and emergency surgery has to be resorted for confirmation of the diagnosis or for relief of obstruction [15,16].

The management of intestinal obstruction due to tuberculosis involves surgery and postoperative treatment with anti-tuberculous therapy [15,17]. The disease, though potentially curable and preventable, still carries a significant morbidity and mortality in Tanzania despite establishment of the National Tuberculosis and Leprosy Programme (NTLP) which was launched by the Ministry of Health and Social Welfare in 1977 as a single combined programme. Factors responsible for this state of affairs are not known. The incidence of tuberculosis has increased dramatically in the last two decades driven by the spread of HIV infection. This increase in incidence has dramatically increased the workload of health care providers and overstretched the existing health systems.

In recent years, our centre has observed a sudden increase in the number of patients with bowel obstruction secondary to intestinal tuberculosis. This observation prompted the authors to analyze this problem. The aim of this study was to describe our experiences in the management of bowel obstruction due to intestinal tuberculosis, outlining the clinicopathological profile, surgical management and outcome of tuberculous intestinal obstruction in our local setting and to identify factors responsible for poor outcome among these patients.

## Methods

### Study design and setting

This was a prospective descriptive study of patients operated for tuberculous intestinal obstruction at Bugando Medical Centre (BMC) in northwestern Tanzania from April 2008 to March 2012. BMC is located in Mwanza city along the shore of Lake Victoria in the northwestern part of Tanzania. It is a tertiary care and teaching hospital for the Catholic University of Health and Allied Sciences-Bugando (CUHAS-Bugando) and other paramedics and has a bed capacity of 1000. BMC is one of the four largest referral hospitals in the country and serves as a referral centre for tertiary specialist care for a catchment population of approximately 13 million people.

### Study population

All patients who were operated for intestinal obstruction at BMC during the period of study and in whom the operative and histopathological findings were suggestive of tuberculosis were consecutively enrolled into the study. Patients who failed to give proper history and those without next of kin to consent for the study were excluded from the study. Patients who failed to consent for HIV infection testing were also excluded from the study.

Preoperatively, all the patients recruited into the study had intravenous fluids to correct fluid and electrolyte deficits; nasogastric suction; urethral catheterization and broad-spectrum antibiotic coverage. Relevant preoperative investigations included packed cell volume, serum electrolytes, urea and creatinine, blood grouping and cross-matching and erythrocyte sedimentation rate (ESR). Patients were also screened for HIV testing using Tanzania HIV Rapid Test Algorithm [18] and CD 4+ count using FACS or FACSCALIBUR from BD Biosciences USA. A determination of CD 4 count was only performed in HIV positive patients. Radiological investigations including X-ray abdomen erect and supine, X-ray chest PA-view were done in all patients. Abdominal ultrasound was also performed in some patients suspected to have associated abdominal collections. Patients presenting in a critical condition were treated with vital system support by: administration of Oxygen, ionotropic support when found hypotensive and oliguric despite adequate fluid replacement. After resuscitation, all patients, under general anesthesia were subjected to exploratory laparotomy through midline incision. They had pre-operative anesthetic assessment using the American Society of Anesthetists (ASA) classification [19] as shown in Table 1. To minimize variability in our study, the assignation of ASA class was performed by one consultant anesthetist adhering strictly to criteria above. Adequate hydration was indicated by an hourly urine output of 30 ml/hour. The operations were performed either by a consultant surgeon or a senior resident under the direct supervision of a consultant surgeon.

**Table 1:** American society of anesthetists (ASA) classification

**ASA class**	**Description**
I	Healthy individual with no systemic disease
II	Mild systemic disease not limiting activity
III	Severe systemic disease that limits activity but is not incapacitating
IV	Incapacitating systemic disease which is constantly life threatening
V	Moribund, not expected to survive 24 hours with or without operation

Intraoperative tissue biopsy was taken for histopathological studies; a portion of the tissue was fixed in 10 per cent formalin; routine processing was done as per standard operative procedures and stained with haemotoxylin and eosin. Presence of caseating granulomas surrounded by epitheloid cells, lymphocytes, plasma cells and giant cells were diagnostic of tuberculosis [20,21]. Post-operatively patients were kept nil orally till return of bowl sounds and at that time nasogastric tubes were removed. Intravenous antibiotics were used for up to one week. The postoperative outcome was monitored; patients in ASA classes IV and V were admitted into intensive care unit after surgery. Final diagnosis and postoperative treatment was dependent on the operative findings and histopathological confirmation. Those found to be tuberculous were started on anti tuberculosis therapy according to the Tanzania National Tuberculosis and Leprosy Programme (NTLP). The anti tuberculosis therapy given included Isoniazid, Rifampicin, Pyrazinamide, Ethambutol and Streptomycin. Data on each patient were entered into a pro forma prepared for the study. The study variables included socio-demographic (i.e. age and sex, level of education, occupation and area of residence), clinical presentation, HIV status, radiological findings, timing of surgical procedure, ASA classification, operative findings and surgical procedure performed. The variables studied in the postoperative period were postoperative complications, hospital stay and mortality. Patients were followed up for a period of twelve months or till death whichever is earlier.

### Definitions of terms

*Acute intestinal obstruction* was considered if the patients had absolute constipation, nausea, vomiting and abdominal distension for 24-48 hours with radiological evidence supporting the clinical presentation.

*Sub-acute intestinal obstruction* was considered if the patients had relative constipation, nausea, vomiting and / or distension for more than 48 hours and the radiological findings were supporting the clinical findings.

*Pulmonary tuberculosis* was considered if the patient had sputum positive for acid-fast bacilli and / or X-ray was revealing pulmonary cavitatory lesion or calcified hilar lymph nodes.

*Elective surgery* that is scheduled in advance because it does not involve a medical emergency whereas an *emergency surgery* is one that must be performed without delay; the patient has no choice other than immediate surgery, if they do not want to risk permanent disability or death.

### Statistical data analysis

The statistical analysis was performed using statistical package for social sciences (SPSS) version 17.0 for Windows (SPSS, Chicago IL, U.S.A). The mean ± standard deviation (SD), median and ranges were calculated for continuous variables whereas proportions and frequency tables were used to summarize categorical variables. Chi-square (χ2) test were used to test for the significance of association between the independent (predictor) and dependent (outcome) variables in the categorical variables. The level of significance was considered as P < 0.05. Multivariate logistic regression analysis was used to determine predictor variables that predict the postoperative complications, hospital stay and mortality.

### Ethical consideration

Ethical approval to conduct the study was obtained from the CUHAS-Bugando/BMC joint institutional ethic review committee before the commencement of the study. Patients were required to sign a written informed consent for the study and for HIV testing.

## Results

### Socio-demographic data

**Figure 1:**
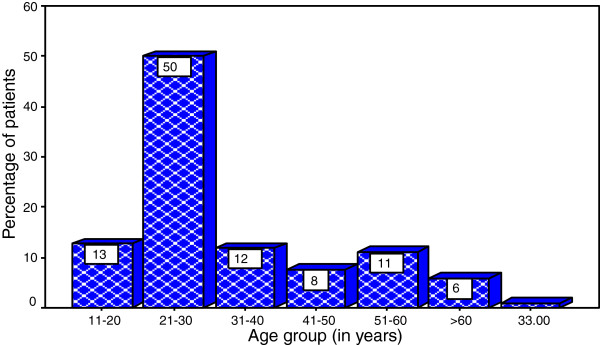
Distribution of patients according to age group.

During the study period, a total of 2643 patients were admitted to our centre and underwent laparotomy for various abdominal conditions. Of these 527 patients underwent laparotomy for bowel obstruction. Out of 527 patients, the underlying cause of obstruction was intestinal tuberculosis confirmed by histopathology in 129 patients. Of these, 11 patients were excluded from the study due failure to meet the inclusion criteria. Thus, 118 patients representing 22.4% of cases (i.e. 118 out of 527 patients) were enrolled into the study. Seventy-six (64.4%) were males and 42 (35.6%) females, with a male to female ratio of 1.8: 1. The age of patients at presentation ranged from 11 to 67 years with a median age of 26 years. The peak age incidence was in the age group of 21-30 years accounting for 50.0% of cases (Figure 1). Eighty-eight (74.6%) patients were aged 40 years and below. Most of patients, 91 (77.1%) had either primary or no formal education and more than 75% of them were unemployed. The majority of patients, 86 (72.9%) came from the rural areas located a considerable distance from the study area and more than 80% of them had no identifiable health insurance.

### Clinical presentation among patients with tuberculous bowel obstruction

The duration of symptoms prior to admission varied between 4 days to 12 months with a median of 8 months. The majority of our patients, 68 (57.6%) had symptoms of more than 6 months duration at the time of presentation. Out of 118 patients, 87 (73.7%) were considered to have primary intestinal tuberculosis and the remaining 31 (26.3%) had secondary intestinal tuberculosis (i.e. associated with pulmonary tuberculosis) with remarkable chest x-rays, past history of pulmonary tuberculosis was positive in only 28 patients (23.7%). Out of these, eight patients were on treatment with anti-tuberculous drugs while fourteen had already taken a complete course of anti-tubercular drugs. The remaining six patients were defaulters.

Sixty two (51.5%) patients presented with acute intestinal obstruction, thirty-four (28.8%) with sub-acute intestinal obstruction, sixteen (13.6%) with signs of peritonism and six (5.1%) with abdominal mass. Abdominal pain was the most common symptom and occurred in all cases (Table 2). In this study, twelve (10.2%) patients had associated pre-morbid medical illness namely diabetes mellitus in 6 patients, hypertension in 4 patients, sickle cell disease and rheumatic heart in 1 patient each respectively. Twenty-five (21.2%) patients were HIV positive. Of these, 8 (32.0%) patients were known cases on anti-retroviral therapy (ARV) and the remaining 17 (68.0%) patients were newly diagnosed patients. Out of 25 patients with HIV, 20 (80.0%) patients were found to have risk factors for HIV infection. Of these, alcoholism [Odds Ratio 14.7, 95% C.I. (7.2-19.3), *p* = 0.011] and multiple sexual partners [Odds Ratio 9.5, 95% C.I. (4.8-14.4), p = 0.001] were found to be independently and significantly associated with increased risk to HIV infection.

**Table 2:** Distribution of patients according to clinical presentation

**Clinical presentation**	**Frequency**	**Percentage**
Abdominal pain	118	100
Vomiting	98	83.1
Constipation	86	72.9
Weight loss	80	67.8
Fever	72	61.0
Abdominal distention	62	52.5
Diarrhea/constipation	25	21.2
Features of peritonism	16	13.6
Abdominal tenderness	82	69.5
Abdominal mass	6	5.1

### Laboratory, radiological and histopathological investigations

Complete Blood Count, Hemoglobin levels and ESR were done in all patients. More than three quarter of the patients had Hemoglobin levels less than 10.0 gm/dl and ESR in the first hour was found ranging between 40-140 mm. Serological investigations for HIV infection revealed that 25 (21.2%) patients were HIV positive. CD4 + count distribution among HIV positive patients ranged from 45 cells/μl to 688 cells/μl with the median CD4 + count of 225 cells/μl. A total of 7 HIV patients (28.0%) had CD4+ count below 200 cells/μl and the remaining patients (72.0%) had CD4+ count of ≥200 cells/μl. Serum electrolytes revealed hypokalaemia and hyponatraemia in 54 and 28 patients respectively. Serum albumin done in 78 patients revealed hypoalbuminaemia in 66 (84.6%) patients. Plain abdominal x-rays (erect/supine) done in all patients revealed multiple dilated loops of bowel with significant air-fluid levels in erect films in 96 (81.4%) patients. Free air under the right dome of diaphragm (pneumoperitonium) was seen in eight (6.8%) patients. Radiography of the chest showed evidence of healed or active pulmonary tuberculosis in 28 (23.7%) patients. Abdominal ultrasound revealed intraabdominal masses in six (5.1%) patients. Barium studies done in 12 (10.2%) revealed one or more of the features like narrowing of distal ileum and ileo-caecal region, matted small bowel. None of our patients had sigmoidoscopy, colonoscopy or Computered Tomography (CT) scan due to lack of these facilities at our centre. Histopathological examination revealed caseating granuloma in 88 cases of resected specimen of intestine only. In 32 patients these granuloma were found in mesenteric lymph nodes as well as intestine. In 8 patients, granulomata were found in parietal peritoneum and serosal tubercles.

### Preoperative anaesthetic assessment

**Figure 2:**
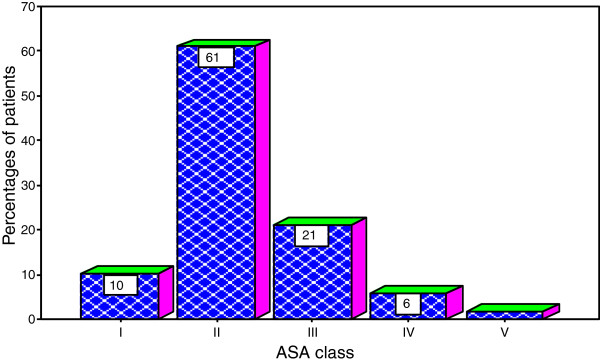
Distribution of patients according to ASA class.

All patients were assessed pre-operatively using the American Society of Anesthetists (ASA) pre-operative grading. The majority of patients had ASA class II accounting for 61.0% of cases (Figure 2).

### Treatment modalities

All the 118 patients had exploration of the abdomen. Sixty-nine (58.5%) patients were operated on emergency bases while 49 (41.5%) patients had an elective surgery. Operative findings of tuberculous intestinal obstruction are depicted in Table 3. The most common area of involvement was the ileo-caecal region in 68 (57.6%) patients. This was followed by the terminal ileum and jejunum in 34 (28.8%) and 12 (10.2%) patients respectively. The colon was involved in 4 (3.4%) patients. The main lesion causing obstruction was intestinal tuberculosis in the hypertrophic form in 86 (72.9%) patients.

**Table 3:** Distribution of patients according to operative findings (N = 118)

**Operative findings**	**Frequency**	**Percentage**
Small bowel strictures (single/multiple)	86	72.9
Bands and adhesions	20	16.9
Bowel strictures and perforation	6	5.1
Ileocaecal mass	4	3.4
Enlarged mesenteric lymph nodes	2	1,7

The right hemicolectomy with ileo-transverse anastomosis was the most common surgical procedure performed in 55.9% of the patients (Table 4). Postoperatively all the patients received antituberculous drugs for a period of one year.

**Table 4:** Distribution of patients according to type of surgical procedures performed

**Type of surgical procedures**	**Frequency**	**Percentage**
Right hemicolectomy with ileo-transverse anastomosis	66	55.9
Segmental bowel resection with end to end anastomosis	28	23.7
Adhesion lysis	20	16.9
Ileo-transverse bypass procedure	2	1.7
Ileostomy	1	1.8
Stricturoplasty	1	1.8

### Treatment outcome

#### Post-operative complications

Forty-four (37.3%) patients had 56 post-complications. Surgical site infection (SSI) was the most common post-operative complication accounting for 42.8% of cases (Table 5). In the present study, the rate of SSI was found to be significantly higher in HIV positive patients than in non HIV patients (*p* = 0.011). Also higher rate of SSI was observed among HIV patients with CD 4 count below 200 cells/μl (*p* = 0.021).

**Table 5:** Distribution of patients according to postoperative complications (N = 56)

**Postoperative complications**	**Frequency**	**Percentage**
Surgical site infections	24	42.9
Enterocutaneous fistula	6	10.7
Wound dehiscence/ burst abdomen	4	7.1
Paralytic ileus	4	7.1
Intraabdominal abscess/ peritonitis	3	5.4
Keloids	3	5.4
Incisional hernia	2	3.6

#### Length of hospital stay

The overall length of hospital stay (LOS) ranged from 1 to 64 days with a median of 24 days. The median LOS for non-survivors was 6 days (range 1-12 days). Patients who had post complications stayed longer in the hospital and this was statistically significant (*p* = 0.011).

#### Mortality

In this study, thirty-four patients died giving a mortality rate of 28.8%. According to multivariate logistic regression analysis, co-existing medical illness (OR = 4.5, 95% C.I. (2.5- 8.9), *p* = 0.001), delayed presentation (OR = 11.3, 95% CI (7.9- 18.4), *p* = 0.023), HIV positivity (OR = 5.9, 95% CI (3.1- 8.9), *p* = 0.002), low CD 4 count (<200 cells/μl) (OR = 7.0, 95% CI (3.9-10.5), *p* = 0.000), high ASA class (OR = 8.1, 95% CI (5.6-12.9), *p* = 0.014), surgical site infection (OR = 1.5, 95% CI (1.1-4.6), *p* = 0.026) were the main predictors of mortality.

### Follow up of patients

Of the survivors, seventy-eight (92.9%) patients were discharged well and the remaining six (7.1%) patients were discharged against medical advice. No patient among survivors in this study had permanent disabilities. Of the 84 survivors, thirty-four (40.5%) patients were available for follow up at three to six months after discharge and the remaining 50 (59.5%) patients were lost to follow up.

## Discussion

In this review, the underlying cause of bowel obstruction was tuberculosis in 22.4% of patients, a figure which is comparable with 21.8% reported by Ali *et al*[22] in Pakistan*.* However, this figure is higher than that observed in many other studies [23-26]. These differences in the rate of tuberculous intestinal obstruction reflect differences in the prevalence and risk factors for developing complications of TB such as bowel obstruction among different study settings. The figures for the rate of tuberculous intestinal obstruction in our study may actually be an underestimate and the magnitude of the problem may not be apparent because of high number of patients excluded from this study.

This study showed that males were slightly more affected than females with a male to female ratio of 1.8:1 which is comparable to the global ratio of 1.5 to 2.1:1 [27]. Some workers report that the disease is more common in males in the western countries while in developing counties the females predominate [28]. We could not find in literature the reasons for this gender differences.

Intestinal tuberculosis, like tuberculosis elsewhere in the body affects the young people at the peak of their productive life [29]. This fact is reflected in our study as the highest age incidence of the patients was in the second and third decades of life and more than seventy percent of our patients were aged forty years and below. This is in accordance with the results of other workers [16,30]. The presentation of tuberculous intestinal obstruction in this age group has serious impacts on the national economy and production, as working and productive class of community is replaced by sick and ill individuals.

Intestinal obstruction resulting from tuberculous has been reported to be more prevalent in people with low socio-economic status [31]. This observation is reflected in our study where most of patients had either primary or no formal education and more than seventy-five percent of them were unemployed. The majority of patients in the present study came from the rural areas located a considerable distance from the study area and more than eighty percent of them had no identifiable health insurance. Similar observation was reported by others [10,31-35]. This observation has an implication on accessibility to health care facilities and awareness of the disease.

The clinical presentation of tuberculous intestinal obstruction in our patients is not different from those in other studies [35,36], with abdominal pain being common to all the patients. The clinical presentation of abdominal TB is usually non-specific [37,38] and, therefore, often results in diagnostic delay and hence the development of complications such as intestinal obstruction [38]. In keeping with other studies [33,35,36], the majority of our patients had symptoms of more than 6 months duration at the time of presentation. The reasons or late presentation in this study may be attributed to the fact that the diagnosis of intestinal TB in its initial stages is usually difficult due to vague and non-specific symptoms as a result patients remain undiagnosed for prolong periods, receiving symptomatic treatment and subsequently present late with complications such acute or sub-acute intestinal obstruction.

In our study, associated pulmonary tuberculosis was found in 23.7% of cases, a figure which is comparable with Baloch *et al*[39]. However, higher figures of associated pulmonary tuberculosis have been reported by others [10,40]. We could not find in literature, the reasons for these differences.

The presence of co-existing medical illness has been reported elsewhere to have an effect on the outcome of patients with tuberculous intestinal obstruction [41]. This is reflected in our study where patients with co-existing medical illness had significantly high mortality rate.

The prevalence of HIV infection in the present study was 21.2%, a figure that is significantly higher than that in the general population in Tanzania (6.5%) [42]. However, failure to detect HIV infection during window period and exclusion of some patients from the study may have underestimated the prevalence of HIV infection among these patients. High HIV seroprevalence among patients with tuberculous intestinal obstruction was also reported by Fee *et al*[43]. This difference in HIV seroprevalence among patients with tuberculous intestinal obstruction reflects differences in the overall prevalence for risk factors for HIV infection in general population from one country to another. High HIV seroprevalence in our study may be attributed to high percentage of the risk factors for HIV infection reported in the present study population.

The clinical picture of tuberculous intestinal obstruction may be complex when tuberculosis occurs with HIV infected patients [44]. HIV infection has been reported to increase the risk of surgical site infection and mortality [45]. In the present study, the rate of surgical site infections and mortality was found to be significantly higher in HIV positive patients than in non HIV patients. Also higher rate of SSI was observed among HIV patients with low CD 4 count (< 200 cells/μl). Patients with AIDS usually have a more severe form of involvement than those who did not have AIDS [37,46]. However, the treatment of bowel obstruction due to tuberculosis in AIDS patients has been reported to be the same as in non-HIV infected patients [47] but multi-drug-resistant tuberculosis is more common in patients with AIDS [48].

Emergency surgical intervention is considered to be the standard treatment of choice for patients with tuberculous intestinal obstruction [36]. In keeping with other studies [10,15,26,35,36], the majority of patients in this study underwent emergency surgical treatment. One of the many factors affecting the surgical outcome in patients with tuberculous intestinal obstruction is time interval between duration of onset of intestinal obstruction and surgical intervention [35,36]. In the present study, the majority of patients were operated more than 24 hours after the onset of illness. Similar observation was reported by other studies done elsewhere [30,36]. Delayed definitive surgery in the present study may be attributed to late presentation due to lack of accessibility to health care facilities, lack of awareness of the disease as a result some patients with tuberculous intestinal obstruction may decide to take medications in the pre-hospital period with hope that the symptoms will abate. It is also possible that some clinicians managing the patients initially may not have considered as a possible diagnosis. In resource-poor countries like Tanzania, difficulties in diagnosis of intestinal TB, patient transfer, and inadequate medical treatment often result in delayed presentation to a hospital [1,2,41].

In agreement with other studies [16,36,49], the ileocaecal region was the most common site of the bowel affected. This is in sharp contrast to other authors who reported the terminal ileum as the most common site of involvement [10,39]. Many studies have been reported that the most common site of involvement of intestinal TB is the ileocaecal region, possibly because of the increased physiological stasis, increased rate of fluid and electrolyte absorption, minimal digestive activity and an abundance of lymphoid tissue at this site [9]. It has been shown that the M cells associated with Peyer’s patches can phagocytes BCG bacilli [50]. The frequency of bowel involvement declines as one proceeds both proximally and distally from the ileocaecal region [9]. In this study, the main lesion causing obstruction was intestinal tuberculosis in the hypertrophic form which is in agreement with Nguyen [51] in Vietnam.

In the present study, the right hemicolectomy with ileo-transverse anastomosis was the most common surgical procedure performed in 55.9% of the patients. This was followed by segmental bowel resection with end to end anastomosis, release of adhesions, bypass surgery, ileostomy and stricturoplasty. Similar surgical treatment pattern was reported by other writers also [15,52,53]. In our study, stricturoplasty was performed in only one patient. This is in contrast to that reported by Akbar *et al*[36] who reported stricturoplasty as the most common surgical procedure performed. Stricturoplasty is a simple procedure originally described by Katariya and his colleagues in Chandigarh India in 1977 [54]. The procedure has become widely popular and is being practiced all over the world. It has also been tried in Crohn’s disease, with the same usefulness [55]. The procedure is simple, quicker, less traumatic and applicable anywhere from pylorus to ileocaecal junction. The procedure can also be undertaken in active lesions [56]. However, this procedure was not popular in our study because the majority of our patients had very extensive local disease with long and multiple strictures and only option was either right hemicolectomy with ileo-transverse anastomosis or by segmental bowel resection with end to end anastomosis. Anti-tuberculous therapy was prescribed in all the tubercular patients postoperatively.

The presence of complications has an impact on the final outcome of patients presenting with tuberculous intestinal obstruction. In keeping with other studies [32,36], surgical site infection was the most common postoperative complications in the present study. High rate of surgical site infection in the present study may be attributed to HIV seropositivity and low CD 4 count.

The overall median duration of hospital stay in the present study was 24 days which is higher than that reported by other authors [20,25,35,36]. This can be explained by the presence of large number of patients with postoperative complications in our study. However, due to the poor socio-economic conditions in Tanzania, the duration of inpatient stay for our patients may be longer than expected.

The overall mortality rate in this study was 22.7% and it was significantly associated with delayed presentation, HIV positivity, low CD 4 count, high ASA class and presence of complications. Addressing these factors responsible for high mortality in our patients is mandatory to be able to reduce mortality associated with this disease.

Self discharge by patient against medical advice is a recognized problem in our setting. Similarly, poor follow up visits after discharge from hospitals remain a cause for concern. These issues are often the results of poverty, long distance from the hospitals and ignorance. Delayed presentation, delayed histopathological confirmation of tuberculous bowel obstruction and the large number of loss to follow up were the major limitations in this study.

However, despite these limitations, the study has provided local data that can be utilized by health care providers to plan for preventive strategies as well as establishment of management guidelines for these patients. The challenges identified in the management of patients with tuberculous bowel obstruction in our environment need to be addressed, in order to deliver optimal care for these patients.

## Conclusion

Bowel obstruction resulting from intestinal tuberculosis is one of the most common abdominal surgical emergencies in our environment and contributes significantly to high morbidity and mortality. Young age at presentation, delayed presentation, poverty and high morbidity and mortality are among the hallmarks of the disease in this region. A high index of suspicion, proper evaluation and therapeutic trial in suspected patients is essential for an early diagnosis and timely definitive treatment, in order to decrease the morbidity and mortality associated with this disease. Factors that were found to be associated with high morbidity and mortality in this study need to be addressed.

## Competing interests

The authors declare that they have no competing interests. The study had no external funding. Operational costs were met by authors.

## Authors’ contributions

PLC participated in study design, literature search, data analysis, manuscript writing, editing and submission of the manuscript. MDM, SEM, PR, HJ and JBM participated in data analysis, manuscript writing & editing. All the authors read and approved the final manuscript.
